# Revealing the spin–vibronic coupling mechanism of thermally activated delayed fluorescence

**DOI:** 10.1038/ncomms13680

**Published:** 2016-11-30

**Authors:** Marc K. Etherington, Jamie Gibson, Heather F. Higginbotham, Thomas J. Penfold, Andrew P. Monkman

**Affiliations:** 1 Department of Physics, Durham University, South Road, Durham DH1 3LE, UK; 2 School of Chemistry, Newcastle University, Newcastle upon Tyne NE1 7RU, UK

## Abstract

Knowing the underlying photophysics of thermally activated delayed fluorescence (TADF) allows proper design of high efficiency organic light-emitting diodes. We have proposed a model to describe reverse intersystem crossing (rISC) in donor–acceptor charge transfer molecules, where spin–orbit coupling between singlet and triplet states is mediated by one of the local triplet states of the donor (or acceptor). This second order, vibronically coupled mechanism describes the basic photophysics of TADF. Through a series of measurements, whereby the energy ordering of the charge transfer (CT) excited states and the local triplet are tuned in and out of resonance, we show that TADF reaches a maximum at the resonance point, substantiating our model of rISC. Moreover, using photoinduced absorption, we show how the populations of both singlet and triplet CT states and the local triplet state change in and out of resonance. Our vibronic coupling rISC model is used to predict this behaviour and describes how rISC and TADF are affected by external perturbation.

## Abstract (web summary)

Knowing the photophysics of thermally-activated delayed fluorescence (TADF) is crucial when designing organic light emitting diodes. Here the authors show that spin orbit coupling in TADF materials is described by a second order vibronic coupling mechanism, and demonstrate the importance of resonance effects to achieve efficient TADF.

## Graphical abstract

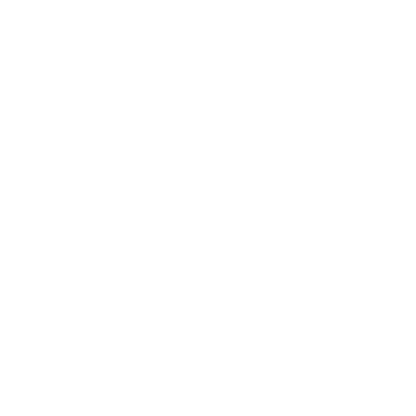

Thermally activated delayed fluorescence (TADF)^1^,^2^,^3^ is a phenomenon of great potential and interest in the organic light-emitting diode (OLED) research community due to the potential for high external quantum efficiencies without the use of heavy metals. In the past, high external quantum efficiencies have been achieved through the use of phosphorescent iridium complexes, where rapid intersystem crossing achieves 100% internal quantum efficiency from triplet states^4^,^5^,^6^,^7^. However, phosphors often suffer from degradation, especially blue phosphors due to intrinsic issues related to the population of higher-lying ^3^dd* metal orbitals^8^,^9^,^10^. Triplet–triplet annihilation is another way of retrieving singlets, at the cost of two triplets for each singlet^11^,^12^,^13^, but these materials are also subject to instability issues^14^. TADF, with its ability to produce an emissive singlet from a triplet state, is the only known mechanism that can achieve a fully fluorescent internal quantum efficiency of 100% (ref. 15).

A typical chemical structure used in effective TADF materials is often that of donor–acceptor (D–A)^16^, or donor–acceptor–donor (D–A–D)^17^ (Fig. 1a), a structure that promotes the formation of intramolecular charge-transfer (CT) states between the donor and acceptor units. Initially, it was proposed that cyclic intersystem crossing within the CT singlet and triplet manifolds gave rise to TADF^1^,^2^, but with the two uncoupled electrons, on the donor and acceptor, being so far apart and in orthogonal orbitals, the spin–orbit interaction between them must be very small, making this scenario unrealistic^18^. However, our recent experimental^19^ and quantum dynamics studies^20^ suggest that the intersystem crossing (ISC) and reverse intersystem crossing (rISC) between ^1^CT singlet and ^3^CT triplet states is a more complicated second order process. This second order process is mediated by vibronic coupling of the ^3^CT to the local exciton triplet (^3^LE) states to allow spin–orbit coupling to the ^1^CT state^18^,^20^,^21^. These results are also in line with the previous findings of Lim *et al*.^22^ and Dance *et al*.^23^

This model predicts that the energy gaps between both ^3^LE–^3^CT and ^3^LE–^1^CT states are critical activation barriers and so the relative energy ordering between the CT states and ^3^LE, along with the magnitude of each barrier controls ISC, rISC and hence TADF efficiency. Here we give spectroscopic evidence from measurements with further theoretical modelling that clearly shows how rISC is affected by the relative energy ordering of the CT and ^3^LE states, substantiating our model and how the predicted changes due to the relative energy ordering affects rISC and TADF. A scheme outlining the ways in which the CT states may move in and out of resonance with a local exciton triplet state is given, controlled by external factors such as host/solvent polarity, and give rise to three distinct types of TADF (Fig. 1b). This work demonstrates the roles played by the ^1^CT, ^3^CT and ^3^LE states in controlling rISC and that all experimental observations are explained by our spin–vibronic coupling mechanism of rISC and thus TADF.

Previous theoretical work on CT states shows that spin–orbit coupling between ^1^CT singlet and ^3^CT triplet states is forbidden^22^. This is because, within the one electron approximation, the spin–orbit operator 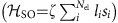 operates upon both the spin magnetic quantum number of the electron and its spatial angular momentum quantum number. Consequently, coupling between singlet and triplet states with the same spatial orbital occupation are formally zero as any change in spin cannot be compensated by a corresponding change in angular momentum, and so total angular momentum is not conserved. For organic TADF emitters, this was confirmed by recent quantum chemical calculations^18^. Therefore:


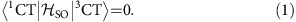


However, ISC between energetically close ^1^CT and a local triplet state, ^3^LE, can efficiently mediate ISC by a spin–orbit charge transfer mechanism with an overlap represented by,


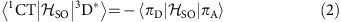


where *π*_D_ and *π*_A_ are the highest filled electronic orbitals of the donor and acceptor respectively.

When the energy gap between ^1^CT and ^3^CT becomes very small (effectively <<1 meV), hyperfine coupling between them may become active as well^24^, but the resulting rISC rate is five orders of magnitude too small to account for the experimental rISC rate. Recently Gibson *et al*.^20^ showed that the mechanism for efficient rISC, involves non-adiabatic coupling between the lowest triplet states, ^3^LE–^3^CT. This coupling, which is much larger than spin–orbit coupling leads to the rapid formation of an equilibrium between the two triplet states via reverse internal conversion. The ^3^CT is then coupled to the ^1^CT state via coupling elements derived from second order perturbation theory, mediated by the ^3^LE state:


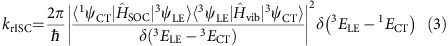


This latter second-order term, which includes the non-adiabatic and spin–orbit coupling terms, is very efficient because of the good vibrational overlap between the almost degenerate initial and final states, ^3^CT and ^1^CT, respectively (Supplementary Methods). Either the donor or acceptor local triplet can take this mediator role^25^.

The ISC rate has also been found to be very sensitive to the position of the local exciton triplet state in polymer:fullerene systems that contain CT states^26^. Here we develop a technique to measure the effect of changing the relative energy separations and orderings between the CT and ^3^LE manifolds to explicitly test our model and clearly show that the second order vibronic coupling mechanism is the driving force for efficient rISC, and hence TADF, in D–A systems.

## Results

### Excited states photoinduced absorption

Figure 2 shows the photoinduced absorption (PIA) for DPTZ-DPTPO2 and the pure phenothiazine donor unit as a reference (PTZ)^27^,^28^,^29^ at room temperature and 20 K in a host matrix of zeonex. In PIA measurements, all excited state populations are sampled, both emissive and non-emissive states. Signals in-phase with the modulation signal relate to fast decaying signals commensurate to the timescale of the prompt CT emission, typically 10 μs (ref. 30), and the out-of-phase signal is generally related to long lived species. It is apparent, through comparison with the reference PTZ spectra that both the in and out-of-phase PIA of the DPTZ-DPTO2 is dominated by the triplet absorption (T_1_→T_N_) of the local phenothiazine triplet at 20 K. This induced triplet absorption of PTZ has been reported at 460 nm in the literature^27^,^28^,^29^ and is confirmed independently in these measurements. The PIA of the dibenzothiophene acceptor has not been reproduced here but in the literature^31^ it is at higher energies than phenothiazine and thus not relevant for these measurements. At room temperature the in-phase PIA spectrum of DPTZ-DPTO2 (Fig. 2a) shows marked differences from the PTZ spectrum with the appearance of a broad induced absorption between 500 and 800 nm. This absorption is attributed to the ^1^CT states as it is in-phase and thus has a similar lifetime to the ^1^CT emission. A broad band similar to this has been observed in photoexcited PTZ solution and assigned to solvated electrons, created from absorption of the stabilized phenothiazine radical anion in solution analogous to the ^1^CT states here^27^,^28^. This broad CT absorption is less prominent at lower temperatures due to the thermally activated nature of the rISC, which reduces the ^1^CT population with decreasing temperature. Thus the ^3^LE and ^1^CT states can be differentiated in the D–A–D system. In the out-of-phase measurement (long-lived species, Fig. 2b) at room temperature, the DPTZ-DPTO2 spectrum is dominated by the broad visible to NIR PIA (450–800 nm) of the long-lived ^3^CT species, identified as having much longer lifetime than the ^1^CT emission. Again, these states are not observed in the pure donor unit.

**Figure 1:**
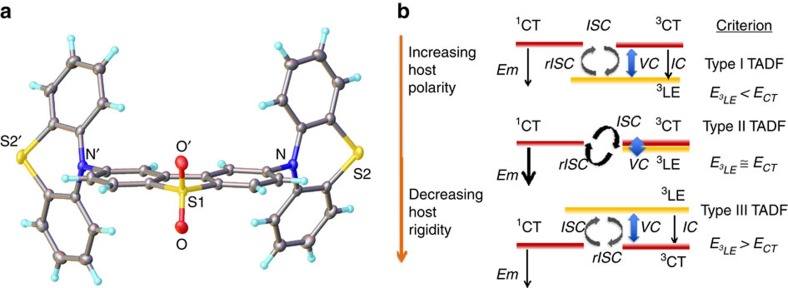
The X-ray structure of DPTZ-DPTO2 and proposed model for the TADF mechanism in this system. (**a**) The donor–acceptor–donor X-ray structure of DPTZ-DPTO2, (2,8-di(10H-phenothiazin-10-yl)dibenzo[b,d]thiophene 5,5-dioxide). (**b**) The proposed model for the TADF mechanisms in DPTZ-DPTO2, which considers the ISC between ^1^CT and ^3^LE based on the inhibited intra CT states ISC and rISC. VC represents the vibronic coupling between states. Type I is a system of high rigidity/low polarity so that ^3^LE is below ^1^CT and Type III is a system of low rigidity/ high polarity where ^3^LE is above ^1^CT, such that in both cases ^3^LE is off-resonance from the CT states. Type II represents the ideal case and the most efficient TADF system where molecular structure and polarity and rigidity controlled energetics are such that ^3^LE is on-resonance with the CT state, giving high efficiency rISC and hence TADF.

The host, zeonex, has a similar polarity to non-polar solvents such as methyl cyclohexane (*ɛ*=2.02) and also has a very high glass transition temperature (375 K) meaning that there is no ‘solvent' reorientation and the CT energy and the molecular structure of the guest are fixed. At this polarity the CT states will lie above the local triplet, found at 2.58 eV, found from previously measured phosphorescence of DPTZ-DPTO2 (refs 16, 19). This places this arrangement as an example of Type I TADF in Fig. 1b.

### Effects of polarity on emission and photoinduced absorption

To investigate the effect of energy ordering of the excited states, by observing the polarity dependence emission and PIA spectra, 20 μM solutions of DPTZ-DPTO2 in toluene (*ɛ*=2.38) and 2-methyl tetrahydrofuran (MeTHF) (*ɛ*=6.97) were studied and compared with films made in the host polyethylene oxide (PEO) which is more polar than zeonex. More significantly, PEO enables us to study polarity dependences in the solid state as it has a temperature dependent dielectric coefficient. At room temperature the dielectric constant of PEO is quoted as *ɛ*=5. However, this polarity is measured at microwave frequencies^32^ but at optical frequencies we observe that PEO has a similar polarity to toluene at room temperature (*ɛ*=2.38) given by the energy position of the DPTZ-DPTO2 emission (Fig. 3a). Furthermore, the glass transition temperature (*T*_g_) of PEO is 220 K, much lower than that of zeonex^32^,^33^,^34^, which causes the dielectric to decrease as the temperature decreases.

Figure 3a shows the effect of polarity on the steady state emission spectra of DPTZ-DPTO2 in toluene and MeTHF (whereas the emission spectra of PTZ remains invariant, see Supplementary Fig. 1) alongside that of DPTZ-DPTO2 films made in zeonex and PEO at different temperatures. The emission peak of DPTZ-DPTO2 in toluene and MeTHF is redshifted compared with that in zeonex by 0.2 and 0.4 eV respectively and the intensity of emission reduces as the emission red shifts, in line with the polarity of the solvents. At room temperature PEO acts very much like the toluene solution and by 150 K it is close to zeonex.

This polarity effect on the photophysics of DPTZ-DPTO2 is further emphasized in the PIA investigated in the aforementioned solvents (Fig. 3b) at concentrations of 10 mM. The in-phase spectra show that the ^1^CT emission dominates and that in toluene it is stronger than in MeTHF, a result mirrored in the steady state PL (Fig. 3a). However, the out-of-phase spectra for both solvent systems are dominated by a broad, long-lived CT state induced absorption. This is attributed to the fact that the energy of the CT states are stabilized in the more polar environments such that they now lie below the ^3^LE which is unaffected by host polarity^30^. This provides an extra decay pathway, internal conversion, from ^3^LE to ^3^CT, since interconversion from ^3^CT to ^1^CT is forbidden, making ^3^CT an effective triplet reservoir. This PIA is identified as the ^3^CT state due to it being dominant only in the out-of-phase measurement, concomitant with it having a much longer lifetime than the emissive ^1^CT state (in-phase signal). This is an example of Type III TADF, Fig. 1b and confirms that the PIA spectra can identify the energetic ordering of excited states in the TADF system.

Figure 4a shows how the properties of the DPTZ-DPTO2 in PEO change as a function of temperature, especially around *T*_g_. The ^1^CT energy (measurement of these energies are detailed in Supplementary Figs 2–6 and detailed in Supplementary Methods) of the molecule blue shifts as the *T*_g_ is approached from above and then stabilizes when the PEO becomes rigid below *T*_g_. With this increase in ^1^CT energy the intensity of the emission also increases before reducing again at low temperatures. Energetically this relates to the ^1^CT energy level shifting from below the ^3^LE state (Type III), passing through resonance at 220 K (Type II) and then increasing further and stabilizing above the ^3^LE state (Type I), as depicted in Fig. 1b. The shift in the ^1^CT energy onset is from 2.50 to 2.60 eV bringing the states into resonance and then out of resonance with the ^3^LE state (again unaffected by temperature). This is shown in the shape of the intensity curve (Fig. 4a). The decrease in intensity at lower temperatures will also be the result of the thermally activated nature of TADF, a phenomenon already well documented in literature^1^,^3^,^35^,^36^. The exchange energy between ^1^CT and ^3^CT in DPTZ-DPTO2 is very small given its near perfect orthogonal D–A–D structure, even in very low polarity medium^19^. Thus throughout the thermal range used here ^1^CT and ^3^CT states will remain nearly isoenergetic. From these measurements it is clear that ^1^CT emission is maximized when the gap between the CT states and the local triplet state (of the donor in this case) is minimized and that rISC and TADF depend critically on these energy gaps.

Figure 4b shows the relative rate of reverse intersystem crossing as a function of temperature obtained from model quantum dynamics^20^ using the energetics reported in Fig. 4a. Each point represents a different quantum dynamics simulation with slightly different energy splitting between the ^1,3^CT–^3^LE states as shown in Fig. 4a. The full details of these simulations are provided in the Supplementary Methods and Supplementary Tables 1 and 2. The results show strong qualitative agreement with the temperature dependence in Fig. 4a, which as expected points to a strong correlation between the rate of rISC and ^1^CT emission intensity. A peak in the rISC rate at 225 K is observed because at this temperature in PEO all three states are isoenergetic. The rate rapidly decays either side of this resonance, but this decay is quicker to higher temperatures, because although higher temperatures should promote TADF, the separation between the ^1,3^CT–^3^LE states exhibits greater increases at temperatures above 225 K, than below. One deviation of note between Fig. 4a,b is that the rISC is almost completely quenched at lower temperature, while the emission intensity is not (Fig. 4a). This deviation arises from the contribution of prompt ^1^CT fluorescence and from reductions in non-radiative decay arising from increased rigidity of the matrix, neither of which are included in the model quantum dynamics simulations.

### Quasi-steady state photoinduced absorption

**Figure 2:**
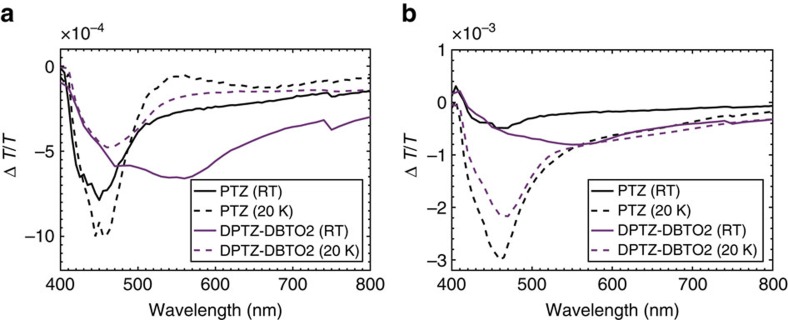
The photoinduced absorption for phenothiazine and DPTZ-DBTO2 in zeonex. (**a**) The in-phase component of the PIA of phenothiazine (black) and DPTZ-DPTO2 (purple) in a zeonex host matrix, measured at room temperature (solid) and 20 K (dashed). (**b**) The out-of-phase component of the PIA of the same materials.

**Figure 3:**
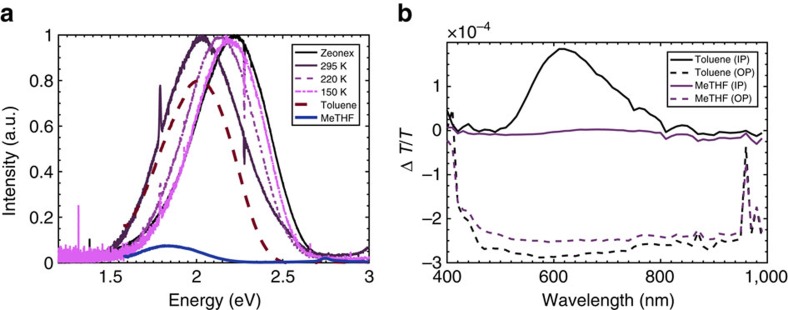
Photoluminescence in solid state and solution with photoinduced absorption in solution of DPTZ-DBTO2. (**a**) Photoluminescence of DPTZ-DPTO2 in a variety of hosts at room temperature unless otherwise stated. The toluene and MeTHF solutions (2 × 10^−5^ M) were excited at *λ*_ex_=400 nm and the samples of ZEONEX and PEO (at 295, 220 and 150 K) were excited at *λ*_ex_=375 nm. The reduction in magnitude for MeTHF is polarity related, while the shift of emission in PEO is related to the glass transition temperature. (**b**) The PIA of DPTZ-DPTO2 in toluene and MeTHF at concentrations of 10 mM. The in-phase spectra (IP) are the solid lines and the out-of-phase (OP) the dashed lines. The broad absorption between 500 and 1,000 nm is related to the ^3^CT states.

The temperature dependence of the PIA of DPTZ-DPTO2 in PEO was measured and is shown in Fig. 5. The in-phase component (Fig. 5a) again shows the increase in intensity of the CT emission, which peaks around *T*_g_. More interestingly, when ^1^CT emission is a maximum at around 220 K, we also observe strong simultaneous ^3^LE_D_ and ^1^CT induced absorption, clearly indicating that at this point the populations of both these states are maximized. Below *T*_g_ the emission falls off and the PIA becomes dominated by the local triplet (^3^LE_D_) induced absorption, similar to that observed in zeonex (Fig. 2). This again is consistent with the fact that at lower temperatures the ^1^CT has shifted above the ^3^LE_D_ state opening up the energy gap between the two, and TADF becomes less efficient as the thermal energy decreases and the long-lived triplet states dominate.

In the out-of-phase signal (Fig. 5b) near *T*_g_, especially 200 K, both triplet absorption and ^3^CT absorption are seen. Again, this is due to the PEO host bringing the CT states into resonance with ^3^LE, that is, Type II arrangement achieved. At 220 K, ^3^CT and ^3^LE_D_ absorption and weak (out-of-phase) emission are observed, a result of TADF being extremely efficient with the large transient ^3^CT and ^3^LE_D_ states population being in thermal equilibrium and producing emissive ^1^CT states. This clearly demonstrates the very small magnitude of the energy barriers between the ^3^LE and CT states that must be reached to achieve efficient TADF and how sensitive rISC is to these gaps. As the temperature is increased and *T*_g_ exceeded the polarity of the PEO increases, the ^1^CT state moves below the local triplet, opening up these gaps and TADF is reduced, shown by the reduction in in-phase emission. These temperature dependent effects are not observed in zeonex as the *T*_g_ is so high that the zeonex is a rigid matrix at all analysed temperatures and so its polarity always remains constant.

### Time-dependent photoluminescence

**Figure 4:**
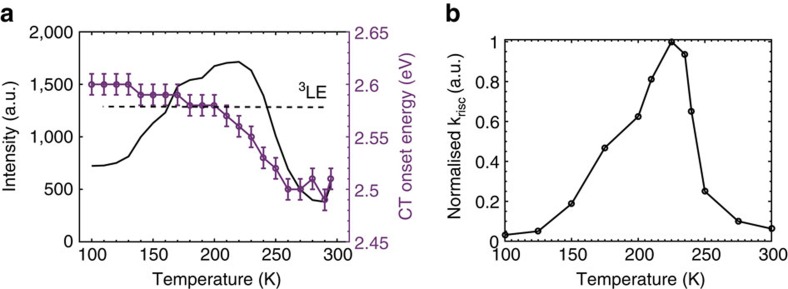
Measured and calculated values of intensity and rISC rate due to change in charge transfer state energy. (**a**) The temperature dependence of the intensity (black line) and CT onset energy (purple circles). The change in CT onset energy plateaus below the *T*_g_, representative of the PEO film becoming rigid. The black dashed line represents the energy of the ^3^LE at 2.58 eV (refs 16, 19), with the peak in intensity apparent as the CT energy crosses resonance. The error bars are indicative of the error in the fit of 0.01 eV for all points, the same error is expected on the ^3^LE energy. (**b**) Relative rate of reverse intersystem crossing as a function of temperature for the D–A complex PTZ-DPTO2. The rates were extracted from the population of the ^1^CT state at 0.5 ns of dynamics simulations initiated from the lowest triplet state. The full details of the model Hamiltonian and the simulations are provided in Supplementary Methods and Supplementary Tables 1 and 2.

**Figure 5:**
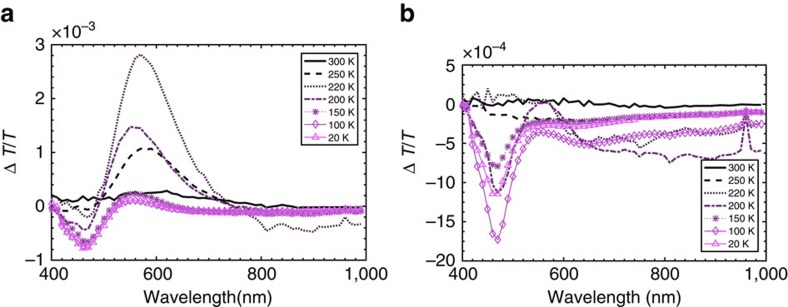
Temperature-dependent photoinduced absorption of DPTZ-DBTO2 in polyethylene oxide. (**a**) The in-phase component of the PIA of DPTZ-DPTO2 in PEO over a temperature range from 300 to 20 K. The resonance peak occurs near the *T*_g_ at 220 K. (**b**) The out-of-phase component of the PIA of DPTZ-DPTO2 in PEO. Between 200 and 220 K there is a crossover between triplet dominated and CT dominated absorption highlighting the resonant point of the system.

**Figure 6:**
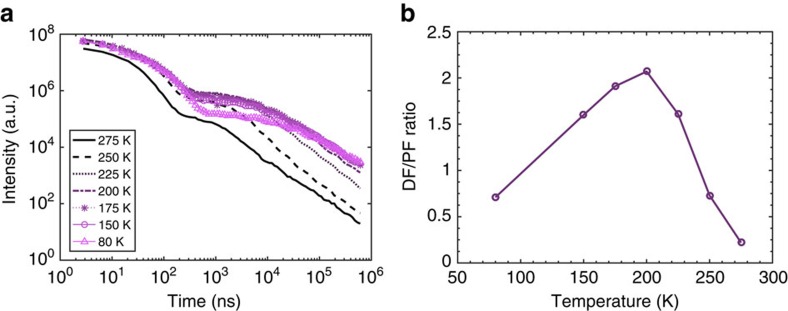
Photoluminescence decay profiles and temperature-dependent DF/PF ratio of DPTZ-DBTO2 in polyethylene oxide. (**a**) The time-normalized decay profiles of DPTZ-DPTO2 in PEO over a temperature range from 275 to 80 K. The maximum rISC rate is observed around the resonance at 225–200 K, which is highlighted from the higher magnitude over which the delayed fluorescence is onset. (**b**) The ratio between the delayed fluorescence and prompt fluorescence of DPTZ-DBTO2 in PEO over a range of temperatures. The prompt fluorescence regime was taken to be between zero time and 250 ns, with the delayed fluorescence regime chosen as being between that and 7.7 μs. The later times have been discounted due to the possible influence of phosphorescence thus these ratios are only relative and not absolute.

Figure 6a shows the transient photoluminescence of DPTZ-DBTO2 in PEO across a range of temperatures. Similar to the resonance observed in Fig. 4, the largest amount of rISC occurs around 200 K, when the CT manifold is in resonance with the ^3^LE. This is indicated by the higher intensity region beyond 10^2^ ns, delayed ^1^CT emission. Below 200 K, the rISC rate is observed to decrease again, with the fall off of the delayed emission component, emphasizing the resonant behaviour of this phenomenon, with the intensity beyond 10^2^ ns at 80 K being similar to the 275 K values. This peak in the DF, rISC and as a result TADF is emphasized in the estimation of the DF/PF ratio, which is shown in Fig. 6b, where the integrated intensity of the delayed fluorescence is divided by the prompt fluorescence. The peak in the values of these DF/PF ratios also occurs around 200 K and is thus in good agreement with the transient spectra (Fig. 6a) and the experimental intensity resonance and theoretical rISC resonance (Fig. 4). In these efficient TADF molecules the non-radiative decay rates are slow and the PF does not change greatly with temperature as a result of this (see first 100 ns of the temperature dependent decay data, Fig. 6a). This means that DF/PF is to good approximation linearly dependent on the rISC rate in these systems. The values for these ratios are shown in Supplementary Table 3. The temperature dependence of the DF for DPTZ-DBTO2 in zeonex has recently been included in the Supplementary Information of work by Dias *et al*.^19^ Here it is seen that the DF monotonically decreases with decreasing temperature and that no resonant behaviour is observed. This is as a result of the non-polar nature of zeonex and its high glass transition temperature.

## Discussion

This work shows that the energy separation between the local triplet state ^3^LE and the CT states of a D–A–D molecule critically controls the efficiency of rISC and hence the TADF process. This supports the second order coupling responsible for rISC^20^, which invokes the role of an intermediate triplet state. We identify this intermediate state, which can be either the ^3^CT or ^3^LE state, depending on the polarity of the host. In both cases, the important factor is the non-adiabatic coupling between them. The presence of an identical PIA peak at 470 nm found in both the PTZ and the D–A–D species analysed here confirms that the lowest energy local triplet resides on the donor. The lower energy, broad induced features are confirmed as induced CT absorption. Furthermore, the rigidity and polarity of the host has a major effect on rISC and hence TADF, also indicates that intramolecular motion of the D–A–D molecule is critical in the TADF process, especially radiative emission^37^. Three distinct regimes for TADF are identified dependent on the relative energetic positions of the CT and ^3^LE states. From these results it is clear that both careful molecular design and host environment are critical in controlling the efficiency of TADF and both must be correct to achieve the desired Type II TADF regime. Most importantly, full experimental verification of our model of second order coupling for efficient rISC^20^ is given, showing the role of all three excited state, ^1^CT, ^3^CT and ^3^LE in rISC and thus TADF. This model is also fully consistent for all CT states, even when ^3^LE is well below the CT manifolds such that enhanced triplet formation is always observed. This model, in which the coupling between the singlet and triplet states is mediated by non-adiabatic coupling between the ^3^CT or ^3^LE states, illustrates the importance of dynamic material design, which explicitly considers the important molecular vibrations that promote this coupling. This understanding may also find application across chemistry, biology and physics to rationalize and manipulate the photophysics of charge transfer/separated states.

## Methods

### Optical characterization

Spectroscopic characterization of the excited state populations of the standard TADF emitter, DPTZ-DPTO2 (ref. 19; Fig. 1a), is performed in different hosts and solvents at a range of temperatures, to resolve which excited states control rISC and investigate how the external environment effects TADF. DPTZ-DPTO2 (2,8-di(10*H*-phenothiazin-10-yl)dibenzo[*b*,*d*]thiophene 5,5-dioxide) is a D–A–D material consisting of phenothiazine donor units (D) and dibenzothiophene-S,S-dioxide acceptor units (A)^19^. Solid state samples were produced by drop casting onto a quartz substrate. The solutions for this process were a 127 mg ml^−1^ solution of the host in toluene mixed in a 1:1 ratio with DPTZ-DPTO2 (concentration 2.7 mg ml^−1^ in toluene). Steady state photoluminescence was measured using a Jobin Yvon Fluoromax-3.

### Quasi-CW PIA measurements

Measurement of the excited state absorption (and emission) spectra^38^, where performed using a 375 nm pump beam (Vortran Stradus 375–60) modulated at 73 Hz, with a continuous laser driven white light source (Energetiq EQ-99X) as the probe. The probe beam was then passed through a Bentham TM300 monochromator and incident on a Si detector connected to the Signal Recovery dual channel 7,225 digital lock-in amplifier that also provides the reference frequency modulation for the pump laser. The temperature dependent emission intensities and hence ^1^CT energies were measured using an Ocean Optics USB4000 spectrograph simultaneously. The intensity of the laser was 305 mW cm^−2^ for all measurements except for DPTZ-DPTO2 in zeonex where 25 mW cm^−2^ was used.

### Time-resolved emission decay

Photoluminescence decays were recorded using nanosecond gated luminescence and lifetime measurements (from 400 ps to 1 s) using a high energy pulsed Nd:YAG laser emitting at 355 nm (EKSPLA). Emission was focused onto a spectrograph and detected on a sensitive gated iCCD camera (Stanford Computer Optics) having sub-nanosecond resolution^3^. The laser fluence used was 160 μJ cm^−2^.

### Quantum chemistry

Quantum dynamics simulations probing the mechanism for efficient rISC were performed using the density operator formalism of the multi-configurational time dependent Hartree (MCTDH) method^39^. Here we adopt a closed quantum system, using the Hamiltonian described in Gibson *et al*.^20^. The energy gap between the ^1^CT–^3^CT states was as calculated in Gibson *et al*.^20^, however the gap between the ^3^LE–^3^CT states was as extracted experimentally below. The full details of the simulations and the model Hamiltonians used is provided in the supporting information.

### Data availability

Data supporting this publication is openly available under an ‘Open Data Commons Open Database License'. Additional meta- data are available at: 10.17634/153015–1 (theoretical) and 10.15128/r2jq085j96k (experimental). Please contact Newcastle Research Data Service at rdm@ncl.ac.uk for access instructions.

## Additional information

**How to cite this article:** Etherington, M. K. *et al*. Revealing the spin–vibronic coupling mechanism of thermally activated delayed fluorescence. *Nat. Commun.* **7,** 13680 doi: 10.1038/ncomms13680 (2016).

**Publisher's note**: Springer Nature remains neutral with regard to jurisdictional claims in published maps and institutional affiliations.

## Supplementary Material

Supplementary Information Supplementary Figures 1-7, Supplementary Tables 1-3, Supplementary Methods and Supplementary References

Peer Review File
