# Supplementary material for: Revealing the spin–vibronic coupling mechanism of thermally activated delayed fluorescence
Source: Nat Commun. 2016 Nov 30;7:13680. doi: 10.1038/ncomms13680 (PMC5141373; doi:10.1038/ncomms13680)
Supplement: Supplementary Information — Supplementary Figures 1-7, Supplementary Tables 1-3, Supplementary Methods and Supplementary References [file ncomms13680-s1.pdf]

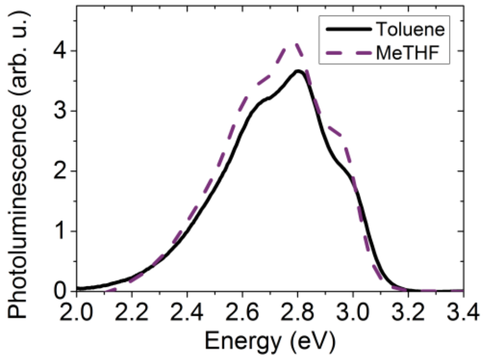

Supplementary Figure 1| The corrected photoluminescence as a function of energy of phenothiazine in toluene and MeTHF solutions at a concentration of 2x10^{-5} M. λ_{ex}= 320 nm.

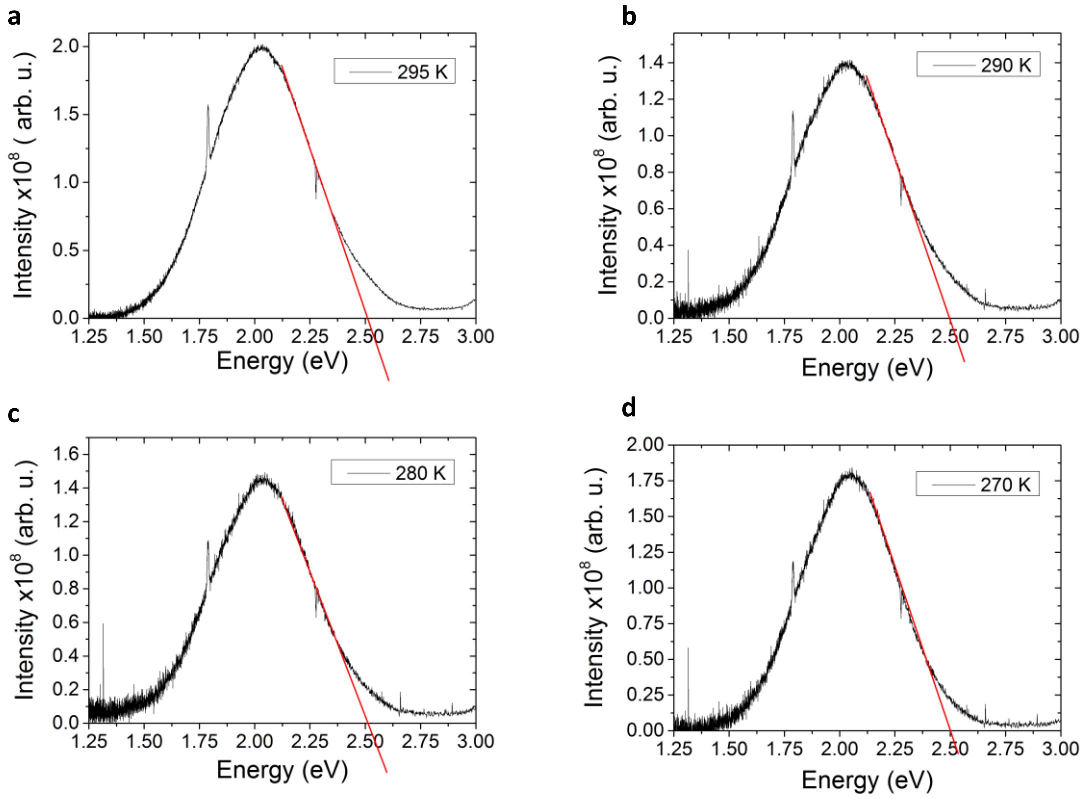

Supplementary Figure 2| Measurement of the onset energy for DPTZ-DBTO2 in PEO as a function of temperature. ***a****, 295 K,* ***b****, 290 K,* ***c****, 280 K and* ***d****, 270 K. The value of the CT onset energy is where the red line crosses the x-axis, which has been set to the baseline of the emission.*

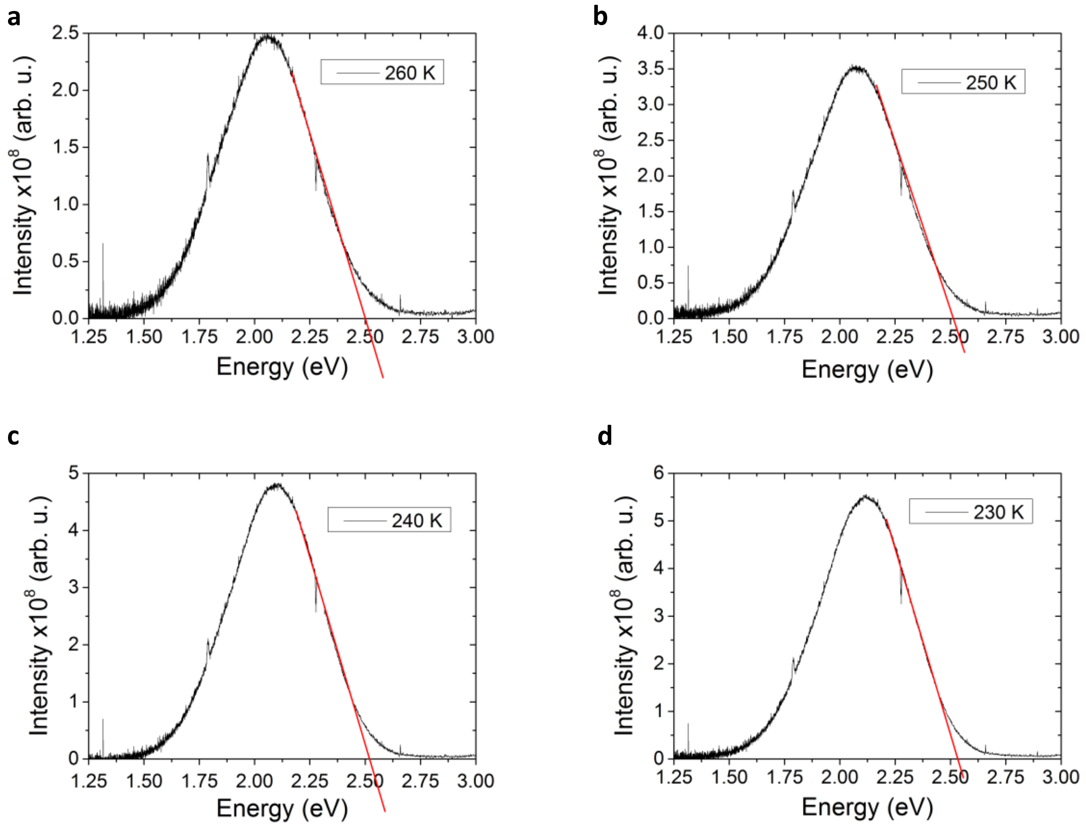

Supplementary Figure 3| Measurement of the onset energy for DPTZ-DBTO2 in PEO as a function of temperature. ***a***, *260 K*, ***b***, *250 K*, ***c***, *240 K and* ***d***, *230 K. The value of the CT onset energy is where the red line crosses the x-axis, which has been set to the baseline of the emission.*

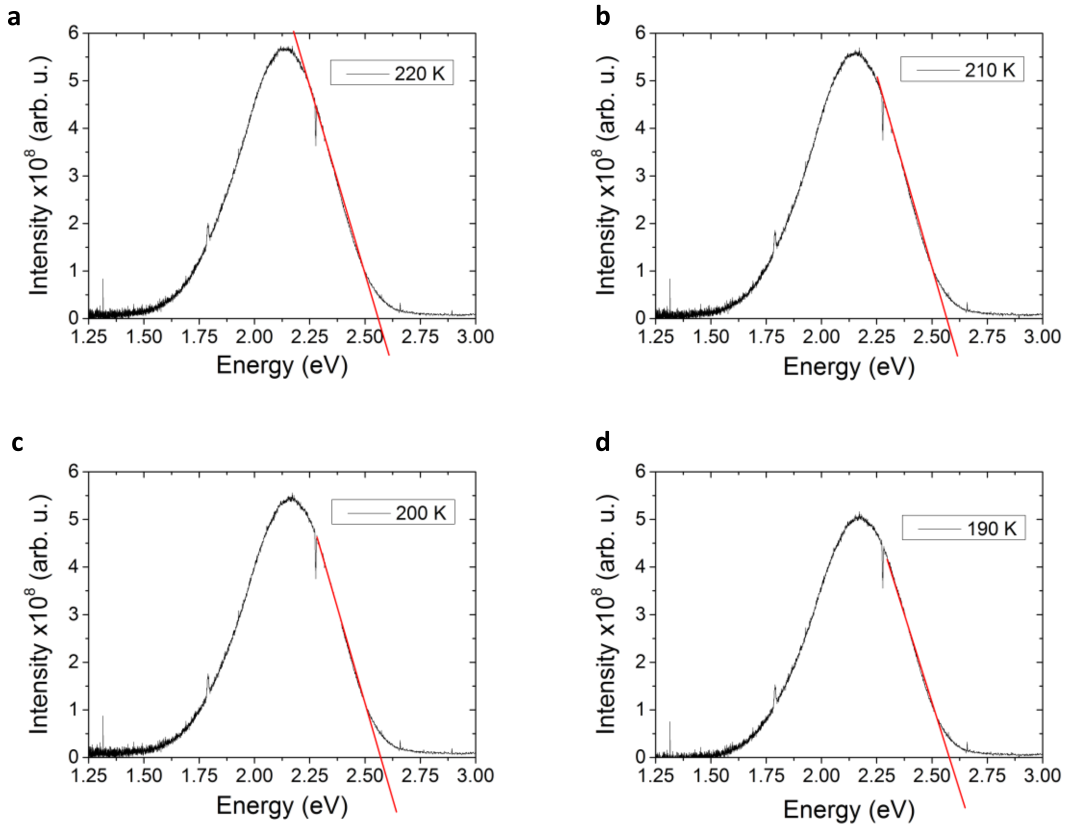

Supplementary Figure 4| Measurement of the onset energy for DPTZ-DBTO2 in PEO as a function of temperature. ***a***, *220 K,* ***b***, *210 K,* ***c***, *200 K and* ***d***, *190 K. The value of the CT onset energy is where the red line crosses the x-axis, which has been set to the baseline of the emission.*

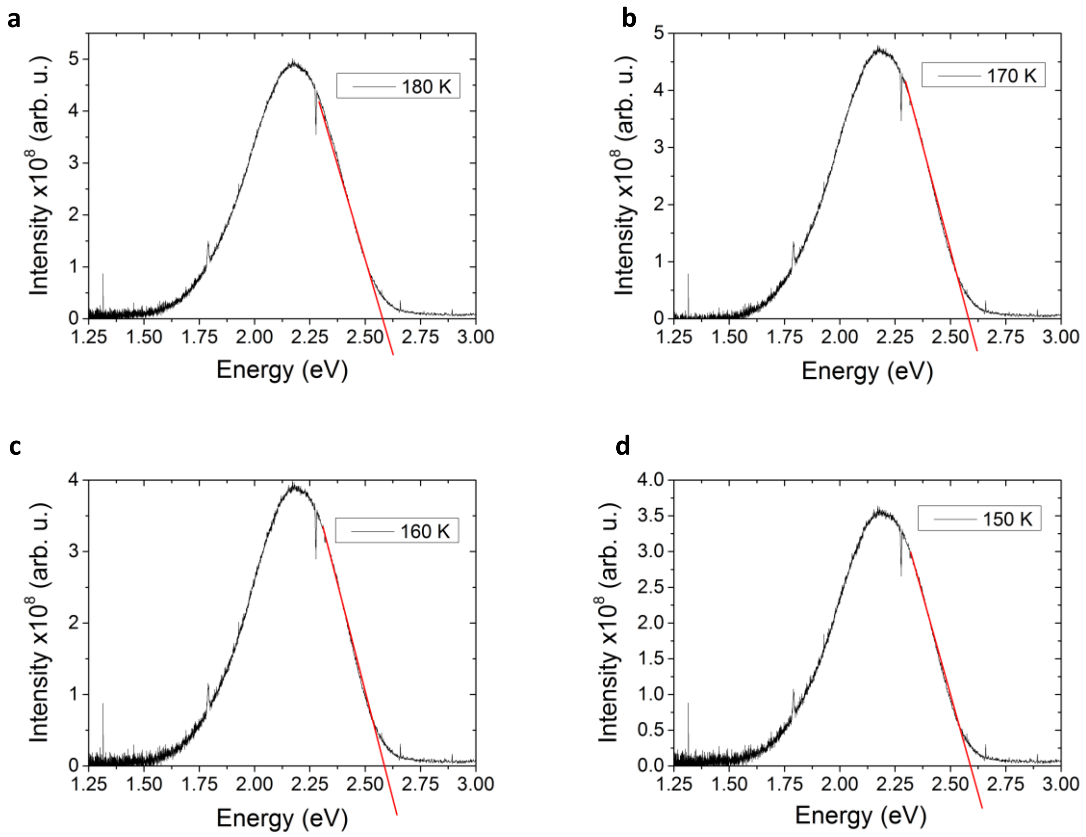

Supplementary Figure 5| Measurement of the onset energy for DPTZ-DBTO2 in PEO as a function of temperature. ***a****, 180 K,* ***b****, 170 K,* ***c****, 160 K and* ***d****, 150 K. The value of the CT onset energy is where the red line crosses the x-axis, which has been set to the baseline of the emission.*

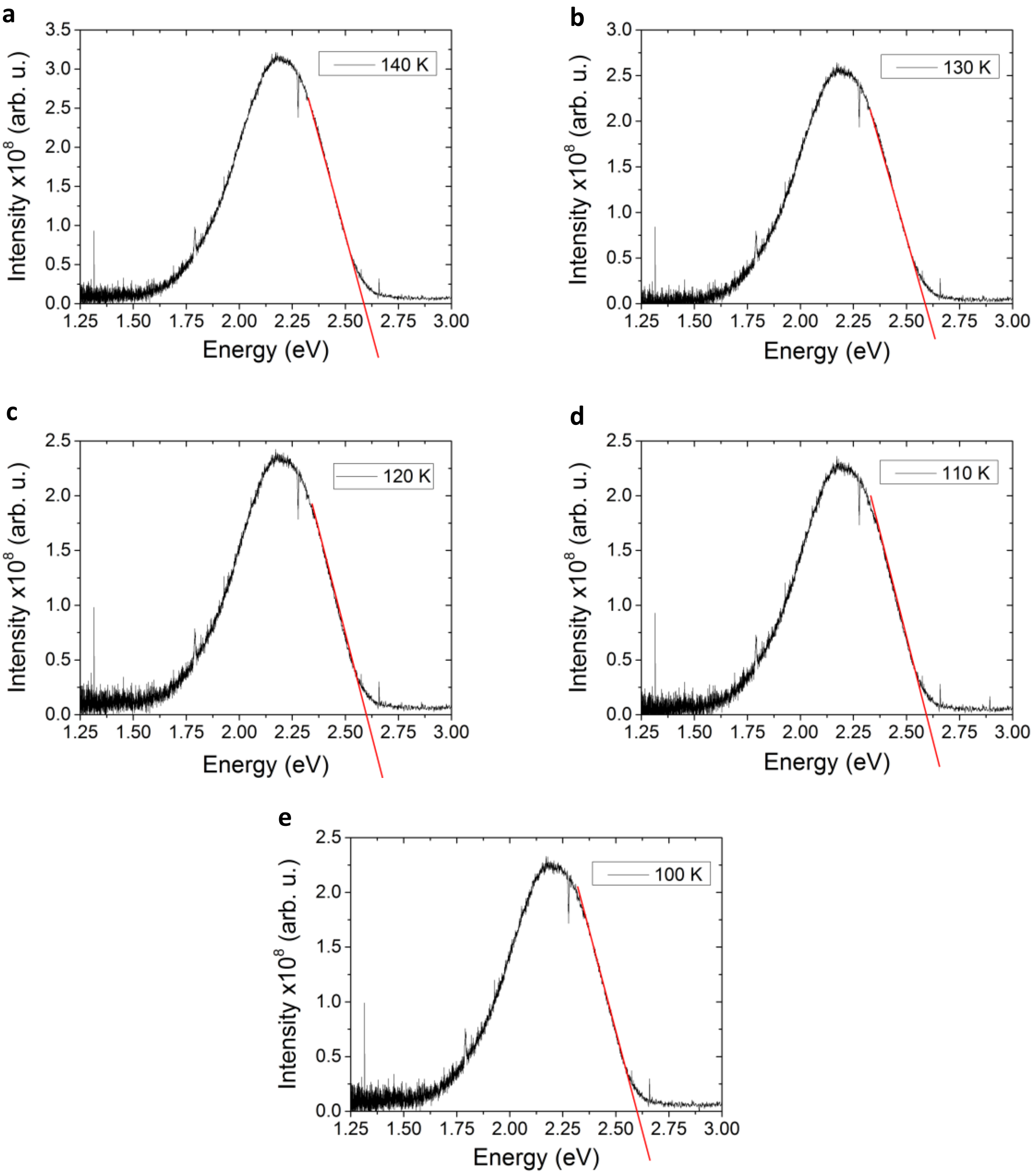

Supplementary Figure 6| Measurement of the onset energy for DPTZ-DBTO2 in PEO as a function of temperature. ***a***, *140 K,* ***b***, *130 K,* ***c***, *120 K,* ***d***, *110 K and* ***e***, *100 K. The value of the CT onset energy is where the red line crosses the x-axis, which has been set to the baseline of the emission.*

Supplementary Table 1| The relative energies of the three excited states.

Simulation	Temperature (K)	^{3}LE (eV)	^{3}CT (eV)	^{1}CT (eV)
1	100	0.00	0.02	0.05
2	125	0.00	0.02	0.05
3	150	0.00	0.01	0.04
4	175	0.00	0.00	0.03
5	200	0.00	-0.01	0.02
6	210	0.00	-0.03	0.00
7	225	0.00	-0.03	0.00
8	235	0.00	-0.03	0.00
9	240	0.00	-0.04	-0.01
10	250	0.00	-0.06	-0.03
11	275	0.00	-0.07	-0.04
12	300	0.00	-0.08	-0.05

Supplementary Table 2| Computational details for the MCTDH simulations within both the wavefunction and density operator formalisms. *Ni is the number of primitive harmonic oscillator discrete variable representation (DVR) basis functions used to describe each mode.* n_i *are the number of single-particle functions used to describe the wavepacket on each state.*

Simulation	Modes	N_i	n_i
1	v1,v11	21	21,61,31
	v23	21	12,12,12
2	v1,v11	21	21,61,31
	v23	21	12,12,12
3	v1,v11	21	21,61,31
	v23	21	12,12,12
4	v1,v11	21	21,61,31
	v23	21	12,12,12
5	v1,v11	21	21,61,31
	v23	21	12,12,12
6	v1,v11	21	21,31,61
	v23	21	12,12,12
7	v1,v11	21	21,31,61
	v23	21	12,12,12
8	v1,v11	21	21,31,61
	v23	21	12,12,12
9	v1,v11	21	21,31,61
	v23	21	12,12,12
10	v1,v11	21	21,31,61
	v23	21	12,12,12
11	v1,v11	21	21,31,61
	v23	21	12,12,12
12	v1,v11	21	21,31,61
	v23	21	12,12,12

Supplementary Table 3| The ratio between the delayed fluorescence and prompt fluorescence of DPTZ-DBTO2 in PEO over a range of temperatures*. The prompt fluorescence regime was taken to be between zero time and 250 ns, with the delayed fluorescence regime chosen as being between that and 7.7 us. The later times have been discounted due to the possible influence of phosphorescence thus these ratios are only relative and not absolute.*

Temperature (K)	DF/PF Ratio
275	0.22
250	0.73
225	1.61
200	2.07
175	1.91
150	1.60
80	0.71

Supplementary Methods

The model Hamiltonian used for the present work is based upon the Linear Spin-Vibronic Model Hamiltonian published in ref.[1] This Hamiltonian has the form:

$$\hat{H} = \begin{pmatrix} \frac{\omega}{2}\left(\frac{\partial^2}{\partial Q_i^2} + Q_i^2\right) + E^{rel}_{^3LE} & \lambda_{Q_i} & E_{SOC} \\ \lambda_{Q_i} & \frac{\omega}{2}\left(\frac{\partial^2}{\partial Q_i^2} + Q_i^2\right) + E^{rel}_{^3CT} & E_{HFI} \\ E_{SOC} & E_{HFI} & \frac{\omega}{2}\left(\frac{\partial^2}{\partial Q_i^2} + Q_i^2\right) + E^{rel}_{^1CT} \end{pmatrix}$$

Where Q_i is the nuclear degree of freedom. In the present model, three nuclear degrees of freedom are included all of which were found, as described in ref.[1] to promote coupling between the ^{3}LE and ^{3}CT states. The relative energies of the three excited states (^{3}LE and ^{1,3}CT) are shown in Supplementary Table 1. All of the other parameters used in the Hamiltonian are unchanged from ref.[1].

The energies of the states are all relative to the ^{3}LE state, which is insensitive to changes in temperature. Consequently, negative energies mean that the CT state is below the ^{3}LE state

The quantum dynamics were performed within a density operator formalism of MCTDH.[2] Here the single particle functions used for the standard wavefunction implementation of MCTDH[3] are replaced with single-particle density operators. Throughout this work we adopt a closed quantum system, this is to say that no dissipative operators are included. The full details the simulations for each Hamiltonian are given below in Supplementary Table 2. All simulations were initiated from the lowest triplet state.

Steady-state emission spectra of DPTZ-DBTO2 and Phenothiazine

The emission spectra of DPTZ-DBTO2 in a variety of hosts has been shown as a function of energy in the main text in Figure 3 a). The representation of the spectra as a function of energy allows for more accurate quantitative analysis of the emission onsets and lineshapes, which is key to the investigation. If conversion back to wavelength is required the conversion factor and theory behind this is nicely presented in a commentary by Mooney and Kambhampati.[4]

Emission spectra, as a function of energy, is also presented for the pure donor, phenothiazine (See Supplementary Figure 1). This highlights that the local singlet emission is not sensitive to the polarity of the host solvent.

Measurement of the charge-transfer state onset energy

As the measurement of the charge-transfer emission was obtained as a function of wavelength, conversion to energy scale needed to be performed first before accurate onset energies can be obtained. This conversion follows the standard correction to both scale and intensity, which is nicely presented in a commentary by Mooney and Kambhampati.[4] By converting the emission spectra into an energy scale (See Supplementary Figures 2-6) the onset can be accurately obtained to within ±0.01 eV.

Supplementary References

1. Gibson, J., Monkman, A. P. & Penfold, T. J. The Importance of Vibronic Coupling for Efficient Reverse Intersystem Crossing in Thermally Activated Delayed Fluorescence Molecules. *ChemPhysChem* **17,** 2956–2961 (2016).

2. Meyer, H.-D. & Worth, G. a. Quantum molecular dynamics: propagating wavepackets and density operators using the multiconfiguration time-dependent Hartree method. *Theor. Chem. Acc.* **109,** 251–267 (2003).

3. Beck, M. The multiconfiguration time-dependent Hartree (MCTDH) method: a highly efficient algorithm for propagating wavepackets. *Phys. Rep.* **324,** 1–105 (2000).

4. Mooney, J. & Kambhampati, P. Get the Basics Right: Jacobian Conversion of Wavelength and Energy Scales for Quantitative Analysis of Emission Spectra. *J. Phys. Chem. Lett.* **4,** 3316–3318 (2013).
