# Supplementary material for: Revealing the spin–vibronic coupling mechanism of thermally activated delayed fluorescence
Source: Nat Commun. 2016 Nov 30;7:13680. doi: 10.1038/ncomms13680 (PMC5141373; doi:10.1038/ncomms13680)
Supplement: Peer Review File [file ncomms13680-s2.pdf]

Reviewers' comments:

Reviewer #1 (Remarks to the Author):

It is becoming increasingly clear that the mechanism of TADF is not as straightforward as the name 'thermally activated delayed fluorescence' might suggest. Operation regimes that do not show thermal activation are a good example. Consequently, I agree that the topic of this manuscript is significant.

This manuscript follows up on prior reports that a third state (a triplet exciton rather than CT state) mediates the interconversion of singlet and triplet CT states. To probe this potential mechanism, the CT state energies are tuned by adjusting the host polarity. It is argued that the TADF rate is maximized when the CT states coincide with the exciton energy.

Overall, I found the work to be provocative and potentially important. But I have some concerns.

1. The intro is peculiar in that it notes the instability of blue phosphorescence to date and then implies that TADF might be stable since it is fluorescent. Of course, all approaches to 100% IQE need to exploit triplets. A better question perhaps is what is the lifetime of these triplets? Presumably the triplet transient lifetime might affect stability. Indeed, determining the maximum rate for TADF should be the key point in this paper.

2. The authors argue that spin orbit coupling is zero for spatially identical singlet and triplet CT states. Is this necessarily true in this DAD molecule? Presumably the 1CT and 3CT states need not be on the same DA combination.

3. One of the most interesting data sets is the thermally _deactivated_ region for T>220K in Fig. 6. But it is a little difficult to understand the physics underlying the DF/PF ratio shown in Fig. 6b. The overall shape is similar to the reverse ISC rate predicted in Fig. 4. Is DF/PF directly proportional to kRISC with no additional temperature dependent factors? The transients in Fig. 6a might have been helpful but they seem to be used only for the normalized magnitude at ~1us. It is also difficult to understand the origin of the transient's overall shape on the log-log plot

4. Why was the transient in Fig. 6a and summary of the DF/PR ratio in Fig. 6b not compared to the non-polar control DPTZ-DPTO2 in zeonex?

5. as an aid to readers, please define 3LE and SOCT when they are introduced

6. It is hard to believe that all TADF materials happen to have a triplet exciton in near resonance to the CT states. Could the authors please comment on this?

Reviewer #2 (Remarks to the Author):

This work resolve around elucidating the energy transfer process in TADF mechanism; the authors have been, through their previous works, advocating the complex non-linear energy transfer process involved in riSC. Here they are using a dedicated system to prove this point. They used a host (PEO) which has a Tg of 220K and by making a series of temperature dependent spectroscopy measurements, they have shown that the efficiency of the TADF process agreed well with the proposed energy alignment (Fig. 1b) as the local triplet state, 3LE, shifts closer/further from 3CT. The shift is influenced by the solvent polarity and the authors have clearly manipulate the polarity

by adjusting the system temperature on either side of Tg.
The results have shown quite conclusively that the TADF is indeed mediated by an efficient vibronic coupling of 3CT to 3LE which is a second order process. On and all I think this is a nice piece of work and should be considered for publication. My only complaint is the rather long and convoluted title which over represented the work within. I will suggest the authors to give this a thought.

Reviewer #3 (Remarks to the Author):

This is excellent work. I recommend acceptance without revision.

Despite the growing importance of thermally activated delayed fluorescence (TADF) emitting compounds in organic light-emitting devices (OLEDs), little is known about the parameters that steer the efficiency of the TADF, save for the small energetic splitting of the lowest singlet and triplet excited states. In particular, the mechanisms that efficiently couple the triplet harvesting state to the emitting singlet state are – so far – not clear.

The authors of the present paper postulate that vibronic interaction between a locally excited (LE) triplet state and the triplet charge-transfer (CT) plays the key role in mediating the intersystem crossing (ISC) from the singlet CT to the triplet CT state and the reverse intersystem crossing (rISC) from the triplet CT to the singlet CT state in donor-acceptor-donor (D-A-D) TADF emitters. To prove this hypothesis, they carried out quantum dynamics studies and devised a very intelligent experimental set-up that allows them to tune the CT states into resonance with the LE state. To this end, the emitter was embedded into a host material with temperature dependent polarity. The results presented in Figure 4 convincingly corroborates the hypothesis that the CT passes through a resonance with the LE state at about 200K. The concomitant quantum dynamics studies include spin-orbit interaction between the triplet LE and CT states, vibronic interaction between the two triplet states as well as hyperfine interaction between the singlet and triplet CT states as possible coupling terms. The outcome of these studies suggests that hyperfine interaction between the two CT states is by far too small and that the combination of spin-orbit and vibronic interaction is required to effectuate the rISC.
The spin-vibronic coupling mechanism of both rISC and ISC in CT states of D-A-D systems has been verified experimentally for the first time. This paper substantially adds to the understanding of the mechanisms underlying (r)ISC in TADF emitters, thus improving the conditions for a rational design of efficient TADF OLEDs.

**Referee 1:** Overall, I found the work to be provocative and potentially important.
**Referee 2:** On and all I think this is a nice piece of work and should be considered for publication.
**Referee 3:** This is excellent work. I recommend acceptance without revision.

**Response:** We thank the reviewers for their efforts and strong support for the publication of this manuscript. Below we address in detail the minor concerns raised by the reviewers.

## Reviewer 1

We have addressed all points and made additions in the manuscript to help improve broader understanding of 'TADF' and thank the referee for highlighting points we may have taken for granted in the original manuscript.

1. The intro is peculiar in that it notes the instability of blue phosphorescence to date and then implies that TADF might be stable since it is fluorescent. Of course, all approaches to 100% IQE need to exploit triplets. A better question perhaps is what is the lifetime of these triplets? Presumably the triplet transient lifetime might affect stability. Indeed, determining the maximum rate for TADF should be the key point in this paper.

**Response:**

The main point we wanted to put forward is that the instability in blue phosphorescence based upon iridium is a result of population of higher lying $^3dd^*$ metal orbitals which cause quenching and degradation of the complex and thus an intrinsic failure preventing commercialization of blue Ir materials. This has been clarified in the introduction. This instability is not present in fully organic TADF emitters. Also with discovery of the resonance behavior shown in this work, krisc rates are increased and triplet lifetimes are decreased to similar values found in iridium. Thus TADF blue does out compete Ir blue.

2. The authors argue that spin orbit coupling is zero for spatially identical singlet and triplet CT states. Is this necessarily true in this DAD molecule? Presumably the 1CT and 3CT states need not be on the same DA combination.

**Response:** The argument holds for both DAD *and* DA molecules. SOC is a short ranged interaction and therefore there has to be some overlap in the orbital electronic density between the initial and final states. However, more importantly, as we highlight, for a 'spin flip' orbital angular momentum has to change during the transition (flip), this facilitates the spin change. When the exchange energy is minimize, as here near 20 meV, the singlet and triplet charge transfer orbitals are identical and spin orbit coupling forbidden. This is true for all systems when j~0. This is supported in X. Chen et al. *J. Phys. Chem. C*, *119*, 9728–9733. (2015) [1]. In this paper the authors found slightly non-zero SOC between $^1$CT and $^3$CT states in D-A molecules (SOC = 0.2 cm-1) due to mixing with a close lying $^3$LE state. However, for D-A-D molecules the SOC actually decreased to 0.08 cm-1. In a recent paper by Nobuyasu et al. [2] it was found that there was very little difference between the D-A and D-A-D molecules in terms of photophysics and device performance. Thus in all cases, the rate of SOC is orders of magnitude to low to yield the experimentally determine $R_{ISC}$ rates, and a wholly new mechanism required to explain efficient TADF, thus the vibronic coupling mechanism discussed here.

3. One of the most interesting data sets is the thermally deactivated region for T>220K in Fig. 6. But it is a little difficult to understand the physics underlying the DF/PF ratio shown in Fig. 6b. The overall shape is similar to the reverse ISC rate predicted in Fig. 4. Is DF/PF directly proportional to kRISC with no additional temperature dependent factors? The transients in Fig. 6a might have been helpful but they seem to be used only for the normalized magnitude at ~1us. It is also difficult to understand the origin of the transient's overall shape on the log-log plot

**Response:** In this case the energy gap is the predominant modification we are making hence the similarity between Fig6b and 4. In these efficient TADF molecules the non-radiative decay rates are slow and the PF changes little with temperature as a result of this, see first 100 ns decay curve data in fig 6 a where PF is virtually temperature independent. This means that DF/PF is to a good approximation is directly proportional to krisc. This has now been added to main text.

The log-log plot is necessary to be able to see all of the decay dynamics in these complicated materials, which occur over many orders of time and intensity. The style of this plot is consistent with previous work by the group.

4. Why was the transient in Fig. 6a and summary of the DF/PR ratio in Fig. 6b not compared to the non-polar control DPTZ-DPTO2 in zeonex?

**Response:** The transient data in Fig. 6a and Fig. 6b for DPTZ-DPTO2 has not been included for the zeonex host as this is found in the SI of a recent paper. [3] This has now been commented on in the main text for reference. The temperature dependence of the DF in zeonex is a monotonic decrease showing clearly no resonant behavior observed in the PEO host.

5. as an aid to readers, please define 3LE and SOCT when they are introduced

**Response:** This has been corrected in the resubmitted manuscript.

6. It is hard to believe that all TADF materials happen to have a triplet exciton in near resonance to the CT states. Could the authors please comment on this?

**Response:** The referee is correct that not all TADF materials have a $^3$LE state close to the CT states (See for example Dias *et al.* [4]). However, TADF emitters that exhibit very efficient rISC, and which are therefore most appealing for applications within OLEDs, do have $^3$LE close to the CT states i.e. S-T gaps of order <20 meV. Either a donor or acceptor local triplet. This point has been added in the text to help show the generality of the mechanism, also helping to answer point 2 of the referee. Showing this necessary requirement is the whole rationale of this paper as synthesising this situation is the most important factor to achieve in the design of efficient TADF molecules.

## Reviewer 2

My only complaint is the rather long and convoluted title, which over represented the work within. I will suggest the authors to give this a thought.

**Response:** We agree, and consequently have modified the title to be:

Revealing the spin-vibronic coupling mechanism of thermally-activated delayed fluorescence


[1] X. Chen, S. Zhang, J. Fan, and A. Ren, J. Phys. Chem. C **119**, 9728 (2015).

[2] R. S. Nobuyasu, Z. Ren, G. C. Griffiths, A. S. Batsanov, P. Data, S. Yan, A. P. Monkman, M. R. Bryce, and F. B. Dias, Adv. Opt. Mater. **4**, 597 (2016).

[3] F. B. Dias, J. Santos, D. R. Graves, P. Data, R. S. Nobuyasu, M. A. Fox, A. S. Batsanov, T. Palmeira, M. N. Berberan-Santos, M. R. Bryce, and A. P. Monkman, Adv. Sci. 1600080

(2016).
[4] F. B. Dias, K. N. Bourdakos, V. Jankus, K. C. Moss, K. T. Kamtekar, V. Bhalla, J. Santos, M. R. Bryce, and A. P. Monkman, Adv. Mater. **25**, 3707 (2013).

REVIEWERS' COMMENTS:

Reviewer #1 (Remarks to the Author):

I support publication of the revised manuscript.
